# Case report of non-healing surgical wound treated with dehydrated human amniotic membrane

**DOI:** 10.1186/s12967-015-0608-8

**Published:** 2015-07-24

**Authors:** Neil H Riordan, Ben A George, Troy B Chandler, Randall W McKenna

**Affiliations:** MediStem Panama Inc., City of Knowledge, Panama City, Republic of Panama; Riordan-McKenna Institute, 801 E. Southlake Blvd., Southlake, TX 76092 USA

**Keywords:** MSC, Amniotic patches, Amnion, Amniotic tissue, Cell therapy, Wound healing

## Abstract

**Introduction:**

Non-healing wounds can pose a medical challenge as in the case of vasculopathic venostasis resulting in a surgical ulcer. When traditional approaches to wound care fail, an amniotic patch (a dehydrated tissue allograft derived from human amnion) can function as a biologic scaffold to facilitate and enhance tissue regeneration and rehabilitation.

**Background:**

Amniotic AlphaPatches contain concentrated molecules of PGE2, WNT4, and GDF-11 which have angiogenic, trophic, and anti-inflammatory effects on tissues that may be useful in enhancing wound healing.

**Aim—case report:**

We present a case of a severe non-healing surgical wound in a 78-year-old male 17 days post right total knee arthroplasty. The full-thickness wound exhibited a mobile flap, measured 4 cm long × 3 cm wide, and showed undermining down to patellar tissue. We treated the wound conservatively for 6 weeks with no evidence of wound healing. Upon failure of the conservative treatment, two amniotic AlphaPatch (Amniotic Therapies, Dallas, TX, USA) were applied to the wound, and the wound healed completely in 10 weeks.

**Methods:**

In the OR, the wound was irrigated with three liters of double antibiotic solution under pulse lavage. Two dry amniotic AlphaPatch (4 cm × 4 cm) were placed over the wound with Acticoat applied on top.

**Results:**

At the two-week follow-up visit (following the incision and drainage of the wound dehiscence and application of the amniotic AlphaPatch), a central scab had formed centrally in the wound dehiscence area. At the four-week follow-up visit, the wound dehiscence area had completely scabbed over with no open areas left. At the eight-week follow-up visit, the scab had just fallen off, and the wound was healing well with immature skin representing the size of a penny. At the ten-week follow-up visit, the wound was completely healed.

**Discussion/conclusion:**

Sterile, dehydrated amniotic tissue AlphaPatches (containing trophic factors known to enhance wound healing) have proven effective in completely healing an otherwise non-healing wound in a 78-year-old male who failed six weeks of conservative wound care treatment.

## Background

Wound healing is a complex process whereby multiple cell types, growth factors, and extra-cellular proteins interact to repair a disruption in the dermal and epidermal layers of the skin. In some cases, the mechanism behind said repair fails to restore the integrity of the injured tissue in a timely manner, delaying the progression of inflammatory, proliferative, and remodeling phases of healing [1]. The resulting chronic, non-healing wound is vulnerable to infection, may cause pain, reduce quality of life, and become a burden on the healthcare system [2]. Non-healing wounds, frequently seen in older patients [3], are associated with certain conditions such as diabetes, obesity, and rheumatoid arthritis, but may also occur following acute trauma or surgical intervention [4, 5]. It is estimated that 1–2% of patients in developed countries will experience non-healing wounds in their lifetime [6], and certain types of chronic wounds are estimated to account for billions of dollars of treatment costs in the United States [3].

An adequate progression of wound healing [1, 7, 8] begins with the secretion of growth factors such as Transforming Growth Factor beta (TGF-β) [9], as well as Fibroblast (FGF), Endothelial (EGF), Platelet Derived (PDGF) and Vascular Endothelial (VEGF) Growth Factors [10]. Neutrophils attracted by PDGF signals clear excess bacteria at the site with the aid of monocytes—later transformed to macrophages. Macrophages regulate the production of TGF-β, which in turn stimulates migration and proliferation of fibroblasts as well as epithelialization [11]. Extracellular matrix and granulation tissue begin forming as fibroblasts secrete fibronectin and collagen precursors concurrently with VEGF-stimulated angiogenesis which carries oxygen and nutrients to the injured site [12]. Finally, the collagen structure in the wound area matures and reassembles into a tighter structure with greater tensile strength. An interruption or delay at any stage of this complex process results in a non-healing wound [2, 13, 14].

Treatments for non-healing wounds include compression therapy, negative pressure wound devices [15], skin grafts [16, 17] and tissue bioengineering [18–20], as well as cell therapy [21] particularly using mesenchymal stem cells (MSC). These cells, known to enhance wound healing, have been broadly studied in clinical trials. Contrary to the early paradigm of cell replacement and differentiation as a therapeutic mechanism of action, evidence is mounting that the *secretions* of the MSC are responsible for their therapeutic effects [22]. These secretions include molecules and extracellular vesicles that yield both local and distant effects. The most important factors present in a conditioned medium of MSC can also be considered protagonists of MSC physiological effects including HGF, TGF-b, VEGF, TSG-6, PGE2 and galectins 1, and 9 [22]. It is important to note that fresh amniotic membranes contain live MSCs while dehydrated amniotic membranes do not. Rather, dehydrated amniotic membranes (as in the case of the AlphaPatch) function as a biologic scaffold to facilitate and enhance tissue regeneration and rehabilitation by way of the said molecules in addition to PGE2, WNT4, and GDF-11 [22].

## Case report

A 78-year-old male with history of prostate cancer and COPD, with a 40-pack-year history of smoking presented for a follow-up visit in the clinic 17 days status post right total knee arthroplasty. During this visit, dermal staples were removed from the surgical incision. The mid portion of the patient’s incision showed some wound dehiscence, representing the same location in which the patient reported a previous sebaceous cyst. Cleocin 300 mg qid was prescribed as antibiotic prophylaxis while Talwin 1-q4prn was prescribed for pain control. The dehisced area was thoroughly cleaned with alcohol and triple antibiotic while the remainder of the wound was Steri-Stripped.

One week later, the patient presented to the clinic for follow-up of the wound dehiscence. Upon physical examination, the wound appeared macerated and white with initial signs of necrosis. The wound was cleaned with alcohol and compressed with Ace bandage. The patient continued to take Cleocin antibiotic regimen.

Eleven days later (35 days post surgery), the patient returned to the clinic to recheck the wound. The wound showed slow healing with no significant drainage. To expedite the healing process, three Monocryl sutures were used to close the wound.

One week later (or 42 days post surgery), the patient returned to the clinic reporting that the three sutures expelled while he flexed his knee during physical therapy. The non-healing wound displayed a sympathetic effusion consistent with vasculopathic venostasis resulting in a surgical ulcer. The wound, however, was not erythematous, hot, or tender to palpation. The patient was scheduled the following day in the OR for drainage of joint effusion with gram stain, knee wound irrigation with pulse lavage, and application of amniotic dry patch to wound.

## Procedure

**Fig. 1:**
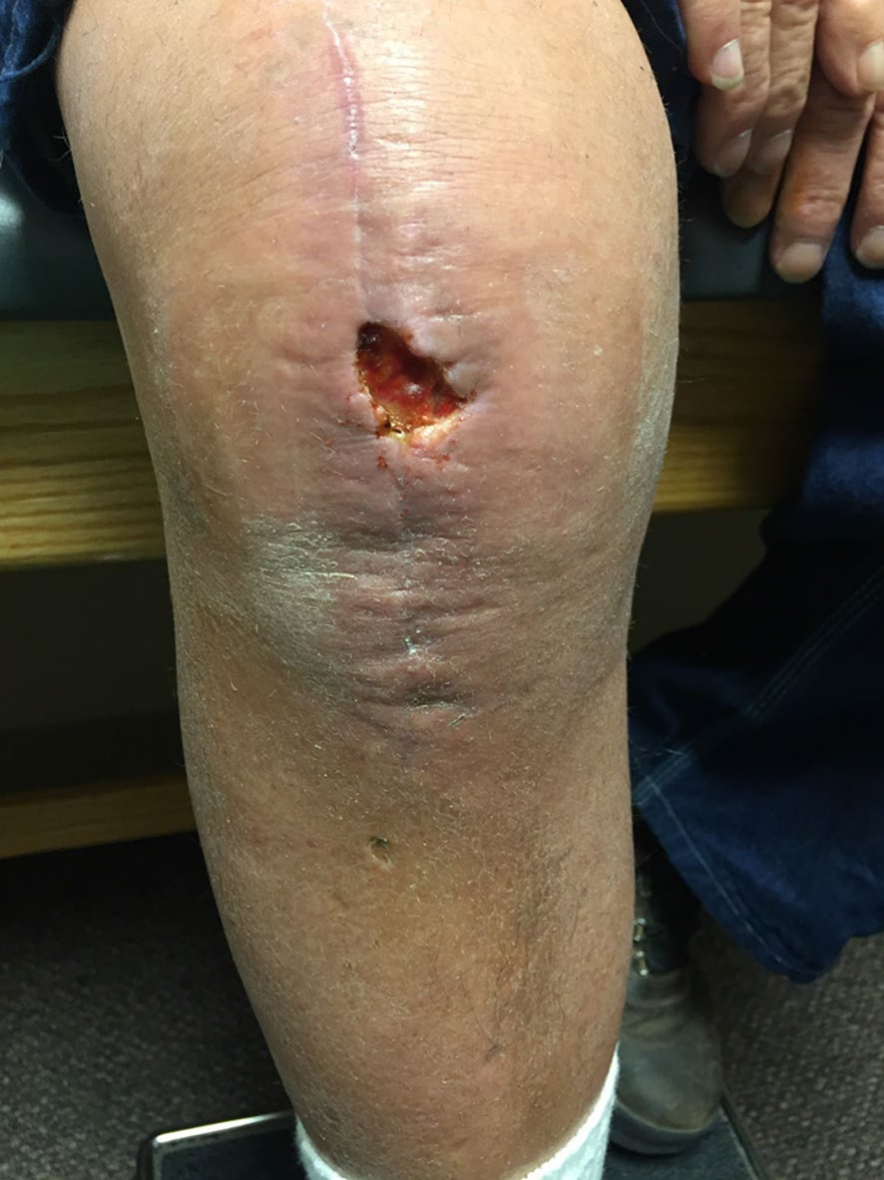
Pre-lavage.

The right lower extremity was sterilely prepped and draped in the usual fashion, roughly 2 months status post total knee arthroplasty. The joint effusion was drained, and 65 cc of bloody synovial fluid was removed from the patient’s right knee. STAT gram stain of the aspirate revealed negative results. The wound was then irrigated with three liters of double antibiotic solution under pulse lavage. Two (4 cm × 4 cm) dry amniotic AlphaPatches (Amniotic Therapies, Dallas, TX, USA) were placed over the wound with Acticoat applied on top. Then 4 × 4’s, Webril, and Ace wrap were applied to the knee. The patient tolerated the procedure well and was transferred to the recovery room in stable condition (Fig. 1).

## Results

**Fig. 2:**
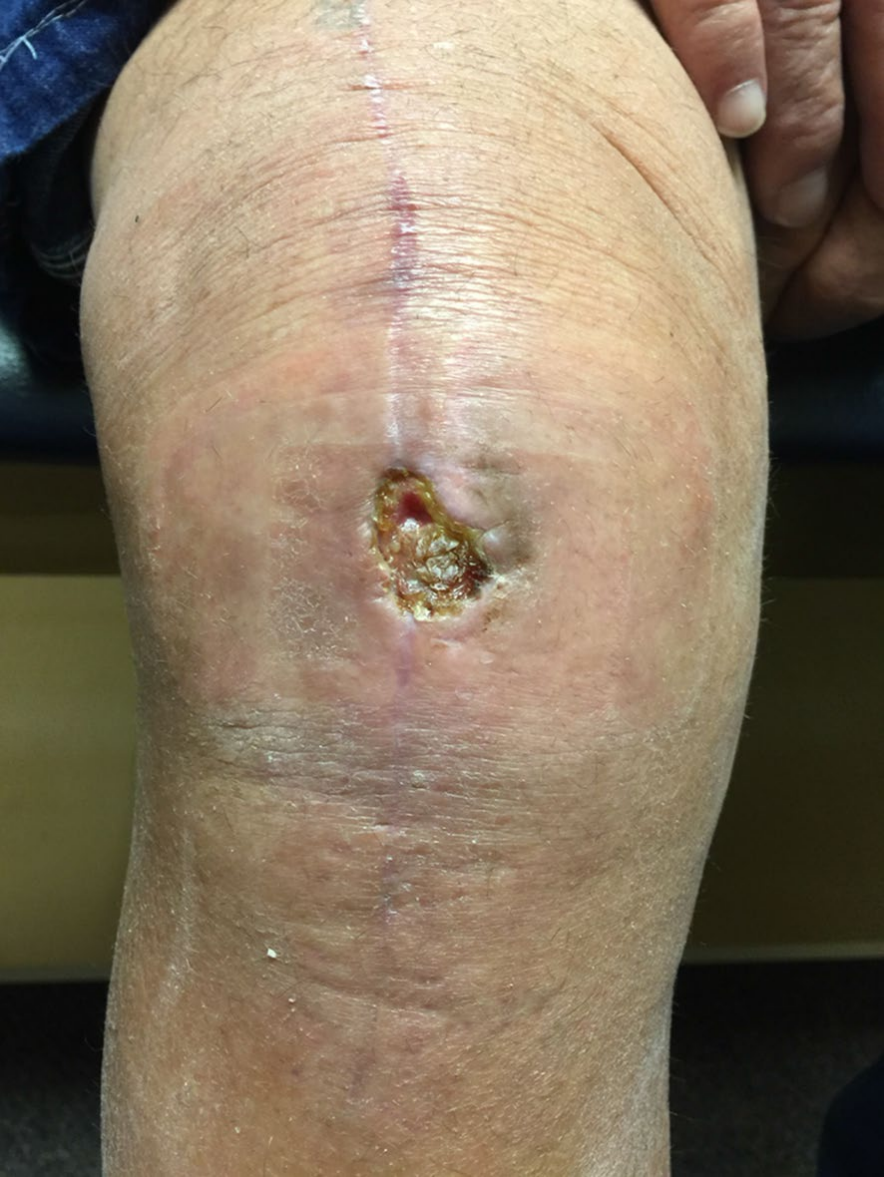
Two weeks post-lavage and amniotic Alpha Patch application.

**Fig. 3:**
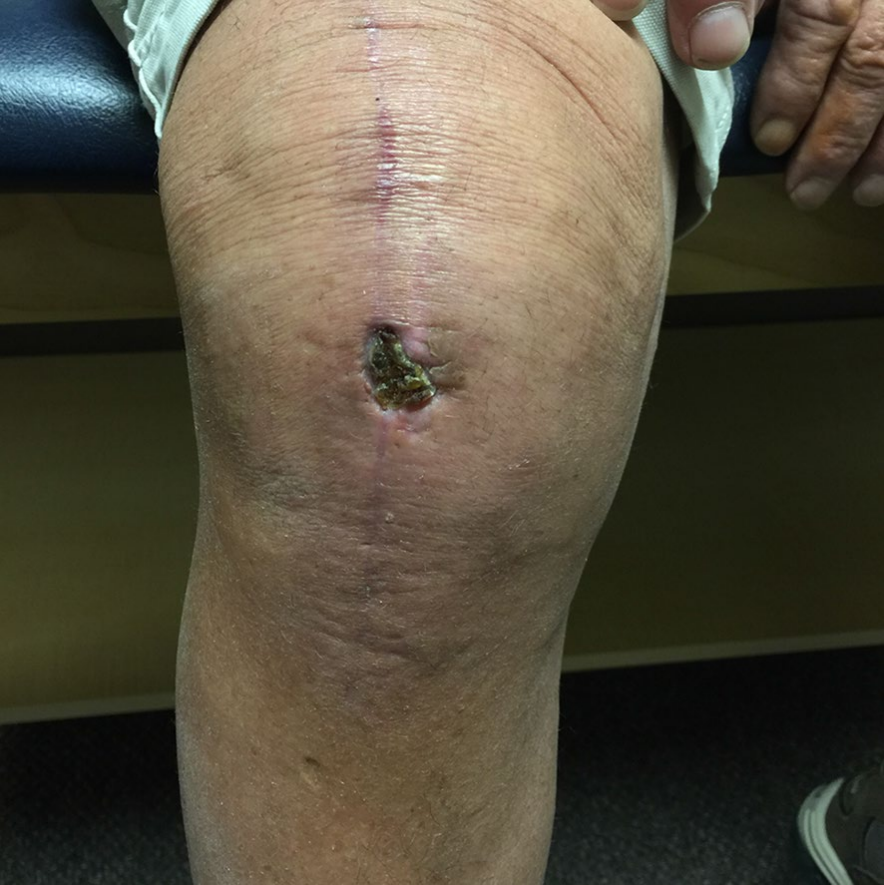
Four weeks post-lavage and amniotic Alpha Patch application.

**Fig. 4:**
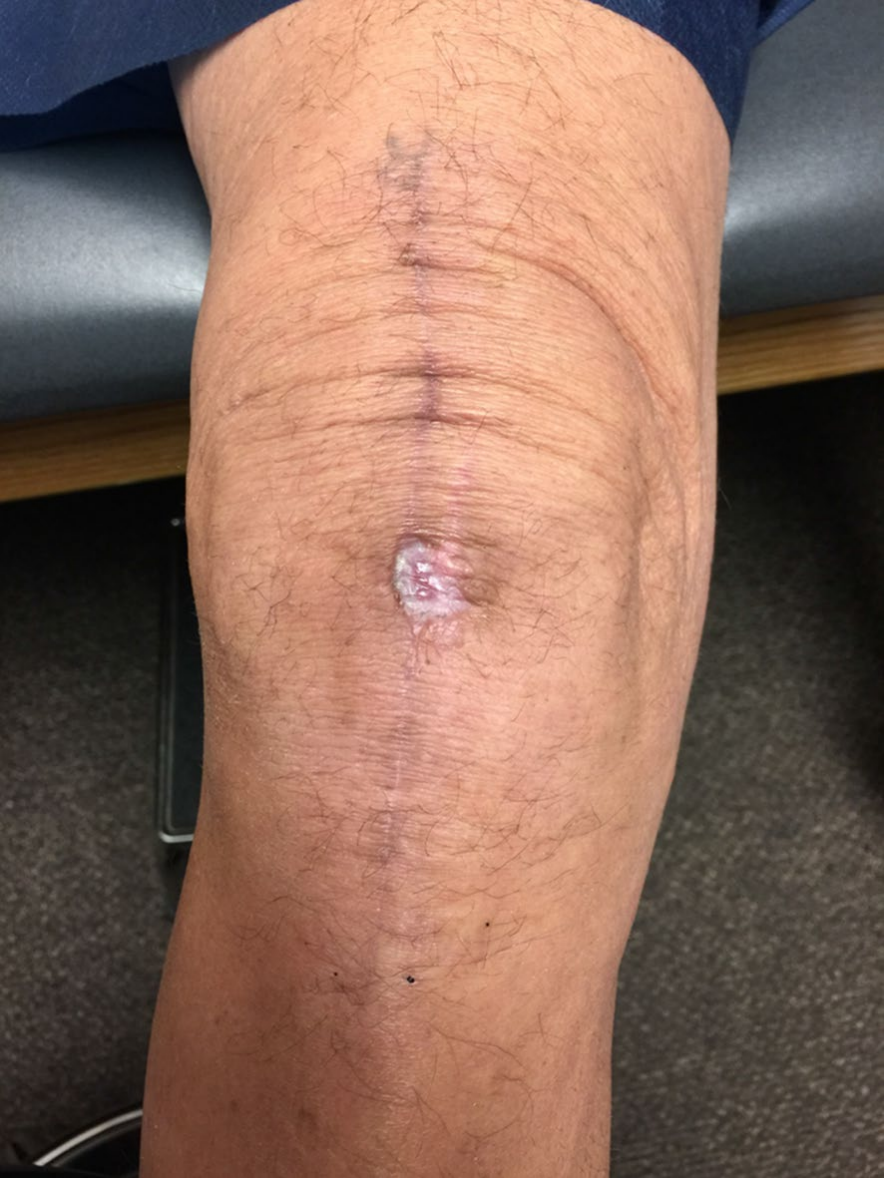
Eight weeks post-lavage and amniotic Alpha Patch application.

**Fig. 5:**
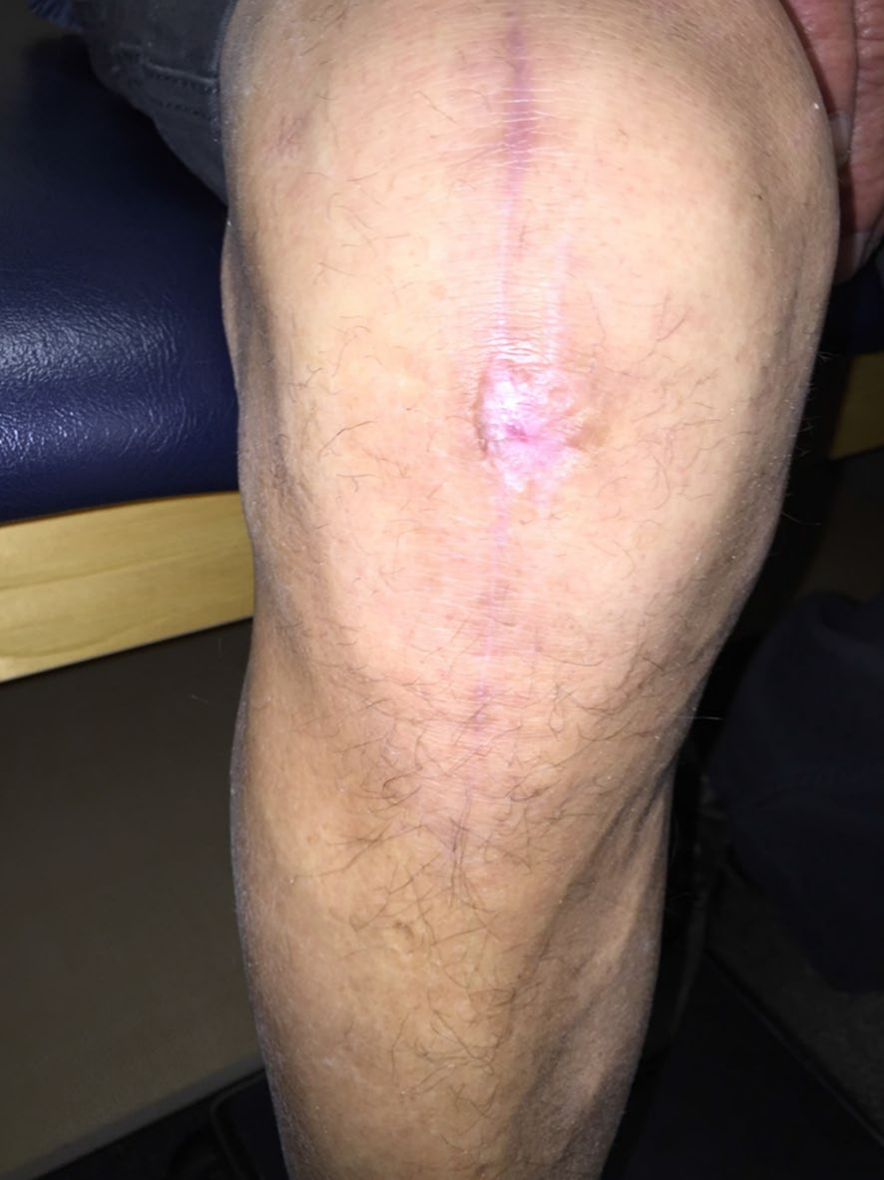
Ten weeks post-lavage and amniotic Alpha Patch application.

At the two-week follow-up visit (following the incision and drainage of the wound dehiscence and application of the amniotic AlphaPatch), a central scab had formed in the middle of the wound dehiscence area (Fig. 2). At the four-week follow-up visit, the wound dehiscence area had completely scabbed over with no open areas left (Fig. 3). At the eight-week follow-up visit, the scab had just fallen off, and the wound was healing well with immature skin representing the size of a penny (Fig. 4). At the ten-week follow-up visit, the wound had completely healed (Fig. 5), patient demonstrated full knee ROM (120° of flexion and 180° of extension), and patient was released from orthopaedic care.

## Discussion

Although the 78-year-old patient demonstrated excellent results in ROM and overall decreased pain levels following right total knee arthroplasty, the non-healing status of the surgical wound at even 42 days status post surgery posed great concern. The vasculopathic venostasis resulting in a surgical ulcer left very little option than to perform dehiscence wound irrigation with pulse lavage and to apply dry amniotic AlphaPatch to the wound.

The amniotic AlphaPatch, a dehydrated amniotic tissue allograft, contains the molecules PGE2, WNT4, and GDF-11. Mesenchymal stem cells (MSCs) secrete PGE2, or Prostaglandin E2, in response to injury. This molecule inhibits fibrosis by way of limiting fibroblast proliferation, migration, collagen secretion, and transforming growth factor (TGF)-induced myofibroblast that can spur fibroblast proliferation [23]. PGE2 also enhances the wound healing process and angiogenesis [24]. WNT4, a protein, drives wound healing by way of wound re-epithelialization and cell proliferation [25]. The creation of new tissue materializes by multiple methods including new blood vessel formation, a critical element for normal wound healing [26]. GDF-11, or growth/differentiation factor 11, has been identified as one of the key molecules propelling the regeneration of skeletal muscle, cardiac muscle, and nervous tissue in aged mice [27].

The two dry amniotic patches applied on the patient’s wound substantially accelerated the wound healing process. The dehisced surgical wound that showed no sign of healing even after 42 days post total knee replacement surgery, demonstrated a central scab formation in the middle of the wound dehiscence area only after 2 weeks of amniotic patch application. After eight more weeks, the wound was completely healed and the patient was released from orthopaedic care to assume high levels of physical activity and activities of daily living. Although more studies are warranted to further substantiate the therapeutic benefits of this treatment, we suggest unreservedly that dehydrated tissue allograft patches derived from human amnion embody a viable and more effective alternative to current traditional means of wound care management.
